# Ca-Mediated Electroformation of Cell-Sized Lipid Vesicles

**DOI:** 10.1038/srep09839

**Published:** 2015-05-07

**Authors:** Fei Tao, Peng Yang

**Affiliations:** 1 Key Laboratory of Applied Surface and Colloid Chemistry, Ministry of Education, School of Chemistry and Chemical Engineering, Shaanxi Normal University, Xi'an 710062, China

## Abstract

Cell-sized lipid giant unilamellar vesicles (GUVs) are formed when lipid molecules self-assemble to construct a single bilayer compartment with similar morphology to living cells. The physics of self-assembly process is only generally understood and the size distribution of GUVs tends to be very polydisperse. Herein we report a strategy for the production of controlled size distributions of GUVs by a novel mechanism dissecting the mediation ability of calcium (Ca) on the conventional electroformation of GUVs. We finely construct both of the calcium ion (Ca^2+^) and calcium carbonate (CaCO_3_) mineral adsorption layers on a lipid film surface respectively during the electroformation of GUVs. It is found that Ca^2+^ Slip plane polarized by alternating electric field could induce a pattern of electroosmotic flow across the surface, and thus confine the fusion and growth of GUVs to facilitate the formation of uniform GUVs. The model is further improved by directly using CaCO_3_ that is in situ formed on a lipid film surface, providing a GUV population with narrow polydispersity. The two models deciphers the new biological function of calcium on the birth of cell-like lipid vesicles, and thus might be potentially relevant to the construction of new model to elucidate the cellular development process.

The vesicles described herein are lipid giant unilamellar vesicles (GUVs), have been used as a model in studies on the basic properties of biomembranes, as the structural foundation for the ideal design of an artificial cell and as an ideal model for biomineralization^1^,^2^,^3^,^4^,^5^. These versatile applications often require the use of high-quality GUVs with few defects which generates a broad interest to develop a highly efficient preparation method for the quick production of GUVs in high yield and narrow polydispersity. Classic and widely employed methods include hydration^6^ and electroformation^6^,^7^,^8^,^9^. The latter is especially attractive since it provides a much higher preparation speed and yield as well as superior quality as opposed to the hydration process. In the electroformation method, GUVs are formed in an aqueous solution by applying a low-frequency alternating electric (AC) field onto a conductive surface with a lipid film coating. Although the detailed mechanism has not yet to be completely elucidated, it is generally believed that the electroosmotic flow of the water induced by this AC field results in the mechanical shearing of the swollen multilayer film^10^. This, in turn, induces vesicle formation through bilayer destabilization and fusion of adjacent vesicles^7^,^8^,^9^. While the pros of such a fusion process include a quick growth of GUVs being facilitated, drawbacks are inevitable as the physics of this self-assembly does not give rise to a strong size selection mechanism for the vesicle radius^11^. Consequently, the size in the resultant GUV population is often very heterogeneous, ranging from a few micrometers up to several hundreds. Naturally, applying some confinements during the growth of the GUVs to suppress the growth/fusion could become a realistic idea to control the size distribution. Up to now, some limited examples have been developed based on the hydration of a micropatterned polymer film^11^ and the design of new electroformation apparatuses to provide in-plane confinement on the growth of vesicles^12^,^13^,^14^. In contrast to these polymer vesicles or instrumentation approaches, we herein for the first time propose a bio-inspired chemical strategy, without remediation on the electroformation setup, that may be designed to achieve the preparation of GUVs with largely improved monodispersities. The principle behind this method also points out a new potential bio-function of calcium during the birth and development of cell-like lipid vesicles.

This strategy, which involves the use of in-situ formed calcium confinements including calcium ions (Ca^2+^) and calcium carbonate minerals (CaCO_3_), is able to mediate the growth of GUVs (Figure 1). Such a mediation process is inspired by biomineralization, renowned for its unique feature involving organic molecule (e.g., biomacromolecule, lipid)-mediated formation of minerals^15^,^16^,^17^,^18^,^19^,^20^,^21^. In contrast, the reverse content as the impact of inorganic minerals on the organic molecules, especially on the self-segregation of these organic molecules has been long overlooked^22^,^23^,^24^,^25^. In the present work, we have paid attention to the calcium element. Calcium (Ca) is one of the necessary nutrients in life^26^,^27^,^28^, and it has been recognized that certain concentration of Ca is beneficial to the growth of organisms through multiple functions such as signaling and catalysis in biochemical reactions^28^,^29^. The classic theories conventionally consider that a particular amount of Ca^2+^ is disadvantageous to the electroformation of GUVs because the screening of the electric field induced by high ionic strength reduces the amplitude of the electroosmotic flow in the system^6^,^7^,^8^,^9^. In nature, the biological regulation on living organisms by Ca^30^,^31^,^32^,^33^,^34^ is a common phenomenon, and could be manifested by spontaneous adsorption of Ca in the form of either Ca^2+^ or calcium mineral as one of the major biominerals around the cell membrane^21^,^28^,^29^. Therefore, inspired by the biological role of Ca when it comes to the self-assembly of bioorganic molecules, our intention was to implant the adsorption of calcium species into the conventional electroformation process of cell-like GUVs. We thus propose a novel adsorption layer theory to guide the efficient parallel electro-production of GUVs with good size homogeneity. In sharp contrast to conventional recognitions, our results find that both Ca^2+ ^and calcium mineral can be adsorbed on a lipid film surface, which finely regulates the growth of GUVs (Figure 1).

## Results and Discussion

### Ca ions-mediated electroformation of GUVs

**Figure 1:**
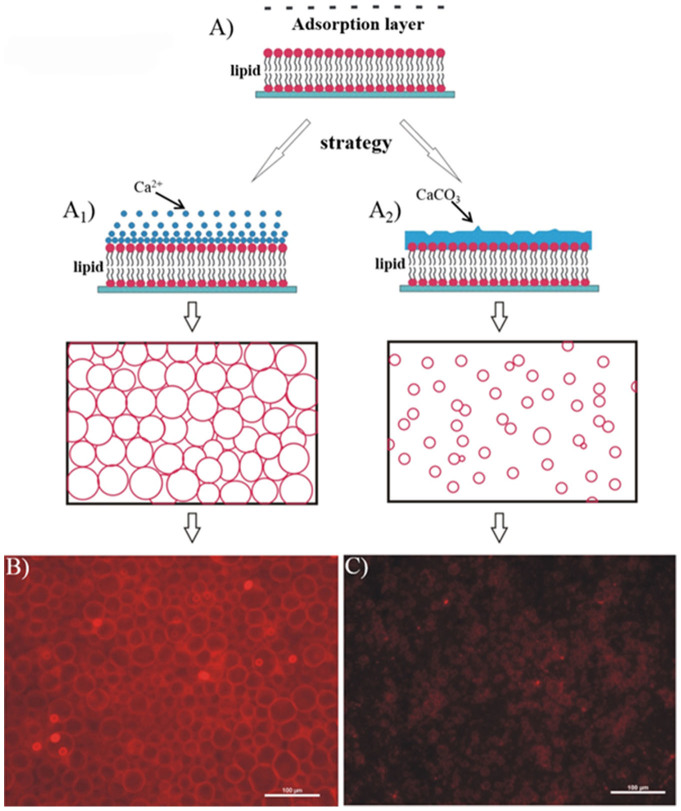
The strategic design for the proposed calcium adsorption layer theory. In (A), the theory could be outlined as two separated models including ions adsorption layer (A_1_) and minerals adsorption layer (A_2_). Both of models resulted in the formation of GUVs with low polydispersity, as reflected by the cartoons just below A_1_ and A_2_. In (B) and (C), the typical experimental evidences to support the conclusion were provided. Image (B) presented the typical fluorescent GUVs prepared according to the model of ions adsorption layer (A_1_), and image (C) presented the typical fluorescent GUVs prepared according to the model of minerals adsorption layer (A_2_).

**Figure 2:**
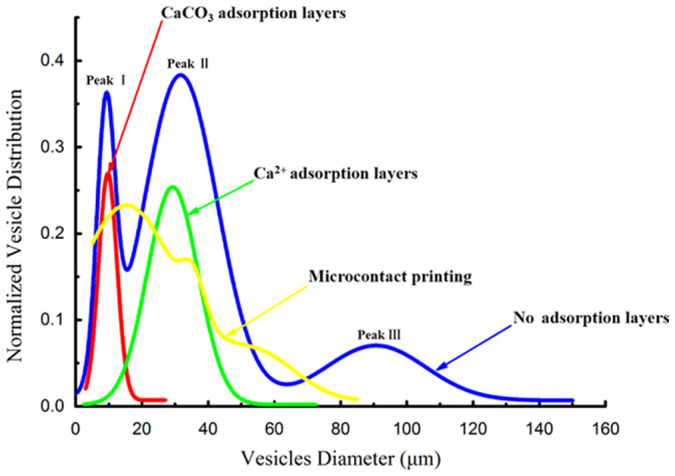
The comparision of size distributions for GUVs prepared by four methods based on different models. Red curve: the model of the minerals adsorption layer; green curve: the model of the ions adsorption layer; deep blue curve: the conventional electroformation without any confinement applied; yellow curve: the reported micro-confined electroformation by combining confinement-free electroformation with microcontact printing-induced lipid film micropatterning.

The first model we developed is based on the molecular interaction between Ca^2+^ and lipid headgroups (Figure 1–A_1_)^35^,^36^,^37^. To mimic the naturally occurring Ca^2+^ adsorption on the headgroups of a cellular lipid membrane, a substantial contact must be taken between the Ca^2+^ solution and the lipid film doped with fluorescent dyes. For this aim, a classic electroformation chamber was first made by assembling an α-phosphatidylcholine (egg-PC) lipid film-coated ITO glass as the bottom, a Teflon spacer as the middle and a pristine ITO glass as the top together with two conductive ITO surfaces facing towards the interior of the chamber (Scheme S1). Before introducing Ca^2+^ in the system, the chamber was filled with an aqueous sucrose solution, and then connected to an AC field function generator by utilizing the two ITO glass plates as parallel flat electrodes (Scheme S1). The peak-to-peak voltage (Vpp) was set as 0.1, and the AC field was applied for 25 min. By using this process as a pre-step, the amphiphilic lipid film coated onto ITO was swollen through the electroosmotic flow of water. The process facilitated stacking of multiple lipid bilayers to form a multi-lamellar, sheet-like film on the surface, exposing phosphate headgroups of lipids outwards at the lipid/water interface for further Ca^2+^ binding^8^,^9^,^38^,^39^. After that, the AC field was switched off, and the Ca^2+^ solution was injected into the chamber through a syringe pump (Scheme S2A), followed by a subsequent exchange of a control solution containing only sucrose to completely flush out the space-filled Ca^2+^ solution in the chamber (Scheme S2B). After that, the chamber was re-connected with the AC field for 25 min with Vpp set as 0.3 (Scheme S2C), followed by repeated Ca^2+^ injections and exchange with a control solution, and another AC field input at 0.5 Vpp for 25 min. The above-mentioned process ensured a substantial adsorption of Ca^2+^ on the lipid film, as reflected by Energy Dispersive Spectroscopy (EDS) mapping on the surface (Figure S1). The fully Ca^2+^-impregnated lipid film was further subjected to a gradually increasing Vpp for vesicle formation without additional injection of Ca^2+ ^solution. We did not observe the obvious formation of GUVs at low Vpp values (e.g., 0.1, 0.3 and 0.5 Vpp). This is reasonably consistent with the classic theory that the adsorbed Ca^2+^ would impede the growth of vesicles due to ionic strength-induced electro-screening^34^. In contrast to this typical recognition, we did observe an effective formation of GUVs in high yield with a few oversized specimens when the voltage increased up to 2.2 Vpp. The size distribution of GUVs at this stage was narrowed (Figure 1B), showing a centered size of approx. 30 μm with a standard deviation being 8 μm (Figure 2). This result is comparable with the recent report^13^ and could be further improved by the second A_2_ model in present work (vide infra). Nonetheless, the ability to control the size distribution by the A_1_ model was superior than the result of the GUVs obtained by conventional electroformation without confinement (Figure 2, Figure S2). A typical size distribution in the conventional electroformation process showed three peaks I-III, which were corresponding to the stages during the GUVs growth ranging from the initial formation of primary GUVs (peak I, stage I) to gradual growth/fusion that further developed into two stages (peak II, stage II and peak III, stage III). We also attempted a reported method to restrict the fusion of GUVs by using in-plane confined lipid film micropatterns^12^. In this process, the micropatterned lipid film was first fabricated on the ITO surface by microcontact printing (Figure S3), and after assembling such an ITO slide into the electroformation chamber, it was found that the area-confined lipid film did not exert obvious restriction on the growth of the GUVs, and the resultant GUVs thus still had chaotic size distributions (Figure 2, Figure S3).

**Figure 3:**
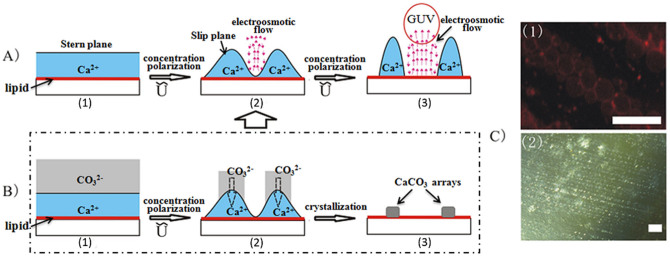
The proposed mechanism for the calcium ions adsorption layer model (A_1_)-mediated electroformation of GUVs and supporting evidences. (A) the proposed schematic process; (B) the CaCO_3_ array formed under AC field by transforming self-segregated Ca^2+ ^barriers to calcite crystal array; (C) the DIC image for the resultant CaCO_3_ array at 0.8 Vpp (C2) and the fluorescent image for the GUVs array corresponding to those CaCO_3_ array (C1). Scale bar = 100 μm.

The improvement of the size distribution of the GUVs by the first model led us to explore this mechanism further (Figure 3). We proposed that the electric field-responsive behavior of Ca^2+^ was the determinative factor for the observed phenomenon. As shown in Figure 3, this key point included three steps. Based on the above-mentioned injection cycles (Scheme S2), the first step was the adsorption and distribution of Ca^2+^ across the lipid film-coated surface through lipid headgroup-Ca^2+^ electrostatic interaction, so that a stable Ca^2+^ Stern plane was formed on the lipid film surface based on an Electric Double Layer (EDL) (Figure 3-A1) (c.f. the detailed description of corresponding physical background can be found in Supplementary Info-2 Part I). The timescale for such entropy-driven adsorption was fully satisfied by the slow injection speed used in the experiment, and the appearance of dense Ca mapping by EDS (Figure S1) was the evidence to support this hypothesis. Steps 2) and 3) were derived from the change of Ca^2+^ Stern plane by the AC field. Upon application of the AC electric field, the Stern plane was replaced with a slip plane according to the Electrokinetic Theory^40^. The resultant non-uniform concentration polarization in the Ca^2+ ^plane caused regular aggregation of Ca^2+^ on the surface. Such segregation of Ca^2+^ on the surface could be fitted by a sine wave function, which consequently induced the circular integral-described distribution of the electroosmotic flow across the surface (Figure 3–A2) (c.f. the detailed description of corresponding physical background in Supplementary Info-2 Part II). The electroosmotic flow, as one determinative factor for the GUV formation, was largely weakened in the Ca^2+^ aggregation areas since the velocity field of the ions dragged the solution to increase viscous drag in the Ca^2+^ aggregation areas. Then, the further elevated Vpp increased the non-uniform concentration polarization that reinforced the electroosmotic flow on the Ca^2+^-deficient lipid film regions to create GUVs (Figure 3–A3)^41^. Overall, with gradually increasing the Vpp, the homogeneous Ca^2+^ adsorption layer was transformed into a patterned Ca^2+^ “barrier” under the AC field, and GUVs finally sprouted from the Ca^2+^-deficient area that was simply the interspaces among the in-situ formed Ca^2+ ^“barriers”. The “barrier” effect subsequently confined the growth and fusion of the GUVs leading to the formation of high-quality and narrow-dispersed GUVs (Figure 1B).

This mechanism could be further supported by the designed experiments. As mentioned above, one key point in this mechanism was the ordered distribution of Ca^2+ ^aggregations on the lipid film surface under the AC field. The conventional optical detection method was hardly utilized to observe these transient distributions in situ. Alternatively, we proposed that the CaCO_3_ crystallization might be an appropriate and simple strategy to display the distribution of Ca^2+^ on the lipid film surface (Figure 3B). In principle, the Ca^2+^-enriched area would produce predominately CaCO_3_ crystals by the combination of Ca^2+^ and CO_3_^2^,^3^,^4^,^5^,^6^,^7^,^8^,^9^,^10^,^11^,^12^,^13^,^14^,^15^,^16^,^17^,^18^,^19^,^20^,^21^,^22^,^23^,^24^,^25^,^26^,^27^,^28^,^29^,^30^,^31^,^32^,^33^,^34^,^35^,^36^,^37^ when supplying carbonate anions to the system. Consequently, the highly dynamic Ca^2+^ segregations could be “frozen” by the crystallization, and such transient Ca^2+^ barriers were actually transcribed to an optically detectable CaCO_3_ array. It is thus suggested that the possible formation of a CaCO_3 _micropatterning on the lipid film surface should shed direct light on the proposed mechanism. For this purpose, the procedures described in Scheme S2 were adjusted by inserting the step of supplying a CO_3_^2−^ (Na_2_CO_3_) solution just after the injection of the Ca^2+^ (CaCl_2_) solution (Scheme S3). After a similar AC field applying and control solution flushing for certain cycles, the expected CaCO_3 _micropatterning could be visualized at a voltage value of up to 0.8 Vpp, showing an ordered array of CaCO_3_ particles under the optical microscope (Figure 3C2). Multiple cycles shown in Scheme S3 were necessary for the formation of the observed CaCO_3_ microarray, because when the Vpp was directly increased to 0.8 without the use of cycles, the ordered CaCO_3 _micropatterning was not observed (Figure S4). The introduction of cycles imparted the system with abundant Ca^2+^ supplement and kinetically slow process approaching a balance state, which ensured substantial relaxation and polarization of the lipid film and complexation with Ca^2+^. The birefringence under the Differential Interference Contrast (DIC) microscope (Figure 3C2) and X-Ray Diffraction (XRD) patterns (Figure S5) indicated a crystalline feature of the micropatterned particles. The grown GUVs also formed an array corresponding to the CaCO_3_ pattern (Figure 3C1), implying that the possible sprouting location for the GUVs was directly related to the interspaces in the CaCO_3_ pattern.

As one control to support the above analysis, the CaCO_3_ micropatterning was not found in the experiments where no AC field was applied regardless whether a lipid film was present or not, as revealed by XRD (Figure S5) and optical inspections (Figure S6 B, C). It was thus suggested that the impact of the AC field was a determinative condition for the formation of CaCO_3_ micropatterning. This was consistent with the suggested hypothesis that the Ca^2+ ^slip plane driven by the AC field induced the organized Ca^2+^ array. Interestingly, we also found that the CaCO_3_ micropatterning formed in a system where the AC field was directly applied onto the blank ITO surface without the lipid film coating (Figure S6 A), even though the resultant patterning was not as good as that formed on the lipid film-coated surface. This phenomenon could be explained by the proposed model. The role of the surface in this model was to provide a base to support certain binding sites with Ca^2+^. This requirement could be easily satisfied on the lipid-coated surface where the hydrophilic phospholipid headgroup could bind with Ca^2+^. This requirement could also be partially fulfilled on the blank ITO surface even without the existence of the lipid film since the Ca^2+^ could interact to some extent with hydroxyl groups on the ITO surface. Such an analysis was in agreement with the observed phenomenon that the resultant array of CaCO_3_ on the blank ITO surface was much sparser than that obtained on the lipid film-coated surface, since the lipid headgroups had a stronger electrostatic attraction towards Ca^2+^ than the hydroxyl groups on the ITO surface. Moreover, the number of lipid headgroups was also much higher than the amount of hydroxyl groups on the ITO surface.

A negative control was further provided to demonstrate the mediation role of the adsorbed Ca^2+^. We found that with the gradual transformation of the Ca^2+^ ions to CaCO_3_ crystals through the above-mentioned crystallization process, the threshold voltage for the growth of GUVs was decreased to 1.3 Vpp, and in addition to the narrow-dispersed GUVs, some over-sized (> 100 μm) GUVs also emerged (Figure S7). This indicated that the barrier effect of the transcribed CaCO_3 _crystal array was worse than that of the initial Ca^2+^-impregnated surface. This reflected the validity of our hypothesis that the Ca^2+^ adsorption layer provided good control over the electroformation of the GUVs, and that the consumption of these Ca^2+ ^ions by forming calcite minerals obviously weakened this control. Overall, the above result refreshed the conventional understanding on the adverse effect of salts on the electroformation of GUVs, which was recently targeted by high-frequency AC field^42^ and hydrogel^38^.

Possible similar role from other cations was also probed by replacing Ca^2+^ with Mg^2+^, Na^+^ and K^+^ respectively. We found that Mg^2+^ and Na^+^ exerted a similar function to Ca^2+^ by narrowing the size distribution of GUVs, while K^+^ did not show obvious capability to modulate such size distribution (Figures S8, S9). Previous reports^35^,^36^ suggest a mechanism in which those metal ions are predominantly bound to the phosphate moieties of lipids. From the solubility coefficient of those four phosphates shown in Table S1, it was reflected that the sequence for the electrostatic interaction of ions with phosphates was: Mg^2+ ^> Ca^2+ ^> Na^+ ^> K^+^. By this sequence, it is thus told that the observed different mediation behavior of the four ions could be attributed to the electrostatic interaction sequence of the ions with phosphates. The biggest solubility coefficient for K^+^ resulted in an extremely weak electrostatic interaction of K^+^ with the phosphate headgroup of lipids. As a result, the electroformation of GUVs was not effectively restrained in the presence of K^+^ and the oversized (> 100 μm) GUVs were observed (Figure S8C). In contrast, the smaller solubility coefficients from Mg^2+^, Ca^2+^ and Na^+^ leaded to the strong electrostatic interaction of these cations with the phosphate headgroup of lipids, and thereby the mediation role from the adsorptive ions on the lipid self-assembly was stringent to control the electroformation of GUVs. Moreover, Mg^2+^ had a much smaller solubility coefficient than Na^+^, which determined the observed phenomenon that the size distribution and average diameter controlled by Mg^2+^ were more narrow and smaller than those controlled by Na^+^.

### CaCO_3_ -mediated electroformation of GUVs

Interestingly, in the second model, we demonstrated by a unique chemical design that the weak controlling ability of the calcite minerals on the GUV electroformation became strong through the construction of a biomineralization layer on a lipid film surface (Figure 1–A_2_). In nature, the inorganic biomineralization process has been closely related to the mediation role of phospholipids with the hydrophilic headgroup pointing outwards^18^,^19^,^20^,^21^. GUVs with functional lipid functionalities can be considered as ideal models to provide not only a confined, organized microenvironment but also an organic matrix for biomimetic mineralization. The present work differentiates itself from previous studies by mainly concentrating on the effect of lipid molecules on the biomineralization^18^,^19^,^20^,^21^, since it has brought to light a new mechanism, namely a reverse process to show a potential biomineral-tuned lipid organization. In this process, the in-situ formed CaCO_3_ mineral layer around the GUV periphery could have a notable effect on the growth of GUVs to offer a narrow-dispersed GUV population (Figure 1C). Another advantage of this process is that the high voltage used in the first model with the ion adsorption layer could be avoided so that the common problems such as the difficulty to control electroformation of the GUVs at high voltage and the risk to encapsulate electro-sensitive molecules in the GUVs during electroformation could be resolved. The corresponding experiment process is described in Scheme S4. In order to obtain the calcite mineral, a precursor for the formation of the CaCO_3_ mineral should be used. We did not directly utilize Ca^2+^ for this purpose, but rather we tried to use the Ca(OH)_2_ colloid as such a precursor. In contrast with the strong responsive behavior of Ca^2+^ in the AC field, a low- frequency (10 Hz) AC field had a weak effect on the Ca(OH)_2_ particles, therefore the interaction between the lipids and the Ca(OH)_2_ colloids was simply attributed to the attractive force between the phosphate group in the lipids and Ca(OH)_2_ without extra AC field-induced particle fluctuation.

**Figure 4:**
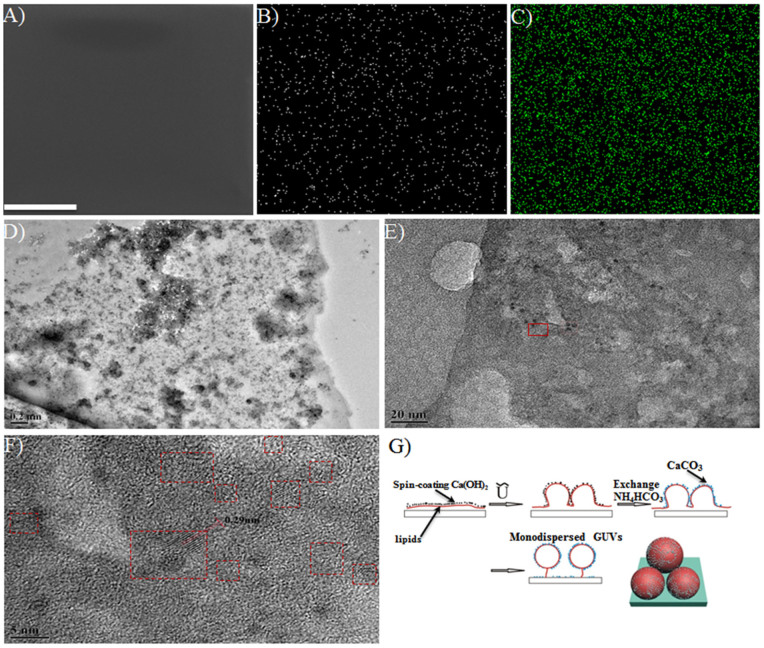
The proposed mechanism for the minerals adsorption layer model (A_2_)-mediated electroformation of GUVs and supporting evidences. (A) SEM photo of the broken GUVs after dropping them onto a bare glass surface; (B) the P element mapping on the surface area shown in (A); (C) the Ca element mapping on the surface area shown in (A); (D, E) TEM images of the lipid membrane fragments of GUVs with different magnifications; (F) the magnified high-resolution TEM image for the selected area indicated by a red rectangle in (E) to show crystal lattices of particles; (G) the schematic process for the formation of GUVs with improved monodispersity by the model of the mineral adsorption layer. Scale bar = 200 μm.

Before assembling the chamber, a colloidal solution of Ca(OH)_2_ particles (1 mg/mL) was spin-coated on the lipid film-coated ITO glass surface. When such a surface was used as the bottom substrate in the electroformation chamber and with the AC field off, an NH_4_HCO_3_ (1 mM) solution was injected into the chamber by the syringe pump to construct the CaCO_3_ mineral adsorption layer on the lipid film surface. Contrary to the precipitation reaction between CaCl_2_ and Na_2_CO_3_, the weak acid-base reaction of the Ca(OH)_2_/NH_4_HCO_3_ combination did not significantly change the ionic strength during the reaction, which was favourable for stabilizing the GUVs. Moreover, the use of weak electrolytes made it possible to also kinetically control a substantial mineralization of Ca(OH)_2_ to form tiny CaCO_3 _particles on the lipid film surface (see below). Both of these features facilitated a well-organized calcite mineral adsorption layer on the surface to constrain the subsequent growth of GUVs. After injection of the NH_4_HCO_3_ solution, the AC field was then introduced into the chamber at a certain Vpp for a given time. The repeated injections of NH_4_HCO_3_ solution and subsequent AC field input were then cycled 3 times with a gradually increased Vpp each time. Narrow-dispersed GUVs were observed (Figure 1C) when the voltage increased to 1.3 Vpp, and they presented a diameter centered at 10 μm with a standard deviation being 3 μm (Figure 2). It can also be clearly seen in Figure 2 that the other two peaks (II and III) representing bigger vesicles due to stage II and III growth/fusion processes were completely diminished by this mineral adsorption layer model. The mechanism behind the experimental result could specifically address this phenomenon (Figure 4G). Upon the formation of primary GUVs in stage I, Ca(OH)_2_ particles were located on these embossments. When the NH_4_HCO_3_ solution was introduced in the system, the adsorbed Ca(OH)_2_ particles could react with the NH_4_HCO_3 _and transform into CaCO_3_, which consequently provided a confined three-dimensional space to further restrict stages II and III (growth and fusion) of the GUVs. In this model, the mineralization step after introducing the NH_4_HCO_3_ solution is important. Without the subsequent mineralization process, we would observe only the appearance of the over-sized vesicles and lipid membrane fragments from the broken vesicles (Figure S10). In this process, Ca(OH)_2_ colloids functioned as the flocculants, and consequently the vesicles could be easily aggregated through the adsorption of Ca(OH)_2 _sol on the vesicle surfaces, which caused a higher probability for fusion among vesicles.

We have further tested the lipid mobility with or without CaCO_3_ mineral layer attached by a standard fluorescence recovery after photobleaching (FRAP) technique, which has been generally accepted as a well-established method to measure such property of lipid membrane. The GUVs without CaCO_3_ mineral layer attached was prepared by exchanging a diluted HCl solution into the chamber where the GUVs with the decoration of the CaCO_3_ mineral layer already electro-formed. With certain incubation time after the solution exchange, the EDS analysis was used to confirm a complete removal of the mineral layer from the GUVs (Figure S11). In order to obtain the solid-supported lipid bilayer membrane for FRAP measurement, an ITO surface was photochemically modified to be hydrophilic by the method developed in our group^43^. And then, the solution containing GUVs with or without CaCO_3_ mineral layer attached was dropped on the hydrophilic surface to induce the rupture of GUVs. Upon the contact of GUVs with the hydrophilic surface, the lipid bilayer membrane from GUVs was laterally spread onto the surface that could be further used as a platform for the measurement of lipid mobility. The FRAP experimental results showed that after the photobleaching, the relative fluorescent intensity of the lipid membrane with the CaCO_3_ mineral layer attached recovered more slowly than that of the lipid membrane without the CaCO_3_ mineral layer attached (Figure S12). The diffusion constant D was 0.258 ± 0.046 μm^2^/s for the lipid membrane with the CaCO_3_ mineral layer removed, while such value was lowered to 0.076 ± 0.008 μm^2^/s for the lipid membrane with the CaCO_3_ mineral layer attached. The different FRAP behaviors indicated that the lipid mobility of the GUVs reduced upon the attachment of the CaCO_3 _mineral layer.

Based on the above analysis, it is easy to imagine that the outer leaflet of resultant GUVs might be decorated with CaCO_3_ particles after the GUV formation (Figure 4G). In order to prove this, the formed GUVs were, after being detached from the ITO slide, deposited onto a glass surface. As the water evaporated, the GUVs came in contact with the hard surface and were broken up to form lipid fragments on the surface. Although such lipid fragments were hardly visualized under scanning electron microscopy (SEM) (Figure 4A), the EDS mapping revealed clearly a dense distribution of the elements P and Ca on the resultant surface (Figure 4B, C), indicating the co-existence of broken vesicles and calcium species on the lipid membrane. A piece of lipid membrane was then subjected to high-resolution Transmission Electron Microscopy (TEM), which clearly showed the accommodation on the lipid membrane of very tiny particles with diameters of several nanometers (Figure 4D, E). The controlled and substantial mineralization under low concentration of reactive species could be responsible for the formation of these tiny crystalline particles. The high-resolution TEM micrograph (Figure 4F) further revealed that the tiny particle had a single crystalline structure with a lattice distance of 0.29 nm. Since the XRD results of the lipid film surface with Ca(OH)_2_ sol spin-coated did not reflect the obvious formation of a brushite phase from calcium phosphate (Figure S13), the observation of a lattice distance of 0.29 nm in Figure 4F could be assigned to the typical crystal plane at (104) for calcite crystals, which were depicted in the proposed mediation process (Figure 4G).

## Conclusions

In conclusion, the present work has led to the discovery that calcium in the form of either cationic Ca^2+^ or CaCO_3_ mineral is favourable for the electroformation of GUVs with low polydispersity. A new theory based on the electrostatic adsorption of calcium onto the phosphate headgroups of lipids in a lipid film has been proposed and interprets the new finding well. We can conclude by two models that under an AC field, the self-assembly behaviour of the lipid molecules in water could be efficiently mediated via the calcium binding. In the first model, the Ca^2+ ^interacts with the lipid film coated onto the ITO surface through the construction of a dynamic adsorption layer on the lipid film. A transient accumulation of Ca^2+^ polarized by an AC field occurs at the water/lipid interface, and the resultant spatial-tuned electroosmotic flow leads to the formation of GUVs with a narrow size distribution. In the second model, the calcite crystals are directly formed in situ onto the lipid film to form a mineral-based adsorption layer at the water/lipid interface. This kind of coverage by CaCO_3_ minerals induces a largely improved polydispersity of the GUVs through spatial confinement around the GUV periphery. Since recent studies have expressed an amazing ability to mediate the self-assembly of organic molecules^22^ and the bioactivity of lipid cells^23^,^24^ by inorganic materials, the present work reveals an unknown role of inorganic species that might be relevant for the origin of life and cellular development in living systems.

## Methods

### Ions Adsorption Layer (A_1_)-Controlled Electroformation of GUVs

To form GUVs, we adapted the electroformation technique invented by Angelova et al.^8^,^9^ First, 20–25 μL of a 2 mg/mL egg phosphatidylcholine (Egg-PC)/chloroform solution containing 4 wt% fluorescein was spread at a constant speed with a micropipette tip on the electrode substrate (ITO-coated glass slide). A self-made chamber for the electroformation was finally enclosed by two ITO-coated glass slides with a Teflon spacer between them and filled with 50 mM control solution (aqueous sucrose solution). The volume of electroformation camber is about 1.5 mL. ITO-coated glass slide with the lipid film coated was always placed as the bottom plate. Then the experiment was conducted step-by-step as follows:

- A sine wave with 0.1 Vpp amplitude and 10 Hz was input into the chamber for 25 min.
- With the AC field being turned off, exchange 1 mM CaCl_2_ solution (5 mL) into the chamber by syringe pump, the injection rate was 6 rmp (0.93 μL/s).
- Exchange control 50 mM sucrose solution (5 mL) into the chamber with a constant injection rate being 6 rmp.
- A sine wave with 0.3 Vpp amplitude and 10 Hz was input into the chamber for 25 min.
- Set step (2), (3) and (4) as one cycle, and repeat this cycle for another 2 times with only the voltage amplitude changing to 0.5 and 0.8 Vpp in step (4) respectively.
- The amplitude increased gradually and stopped to increase until the GUVs could be observed. The final voltage amplitude was around 2.2 Vpp.
- Reduce the frequency to 5 Hz and Vpp to 0.5 for releasing GUVs from ITO surface.

### Fabrication of CaCO_3_ Array on the Lipid Film Surface to Prove the Validity of the A_1_ Model We Built

The experimental process is similar to the above-mentioned procedures except the step as exchanging Na_2_CO_3_ solution was inserted after exchanging CaCl_2_ solution:

- A sine wave with 0.1 Vpp amplitude and 10 Hz was input into the chamber for 25 min.
- With the AC field being turned off, exchange 1 mM CaCl_2_ solution (5 mL) into the chamber by syringe pump, the injection rate was 6 rmp (0.93 μL/s).
- Exchange 1 mM Na_2_CO_3_ solution (5 mL) into the chamber by syringe pump with a constant injection rate being 3 rmp (0.46 μL/s).
- A sine wave with 0.3 Vpp amplitude and 10 Hz was input into the chamber for 25 min.
- With the AC field being turned off, exchange control 50 mM sucrose solution (5 mL) into the chamber with a constant injection rate being 6 rmp.
- Set step (2)–(5) as one cycle and repeat such a cycle for another 2 times with only the voltage amplitude changing to 0.5 and 0.8 Vpp respectively in step (4).

### Minerals Adsorption Layer-Controlled Electroformation of GUVs

Before electroformation, 0.8 mL 1 mg/mL Ca(OH)_2_ sol was spin-coated at 400 rpm on ITO slide with egg-PC lipid film coated. The spin-coating was repeated twice, and then the chamber was assembled by using the spin-coated ITO slide, Teflon and pristine ITO as the bottom electrode, the spacer and the top electrode respectively. After the chamber was filled with 50 mM control solution (aqueous sucrose solution), the following procedures were performed:

- A sine wave with 0.1 Vpp amplitude and 10 Hz was input into the chamber for 25 min.
- With the AC field being turned off, exchange 1 mM NH_4_HCO_3_ solution (5 mL) into the Chamber with a constant injection rate being 6 rmp.
- A sine wave with 0.3 Vpp amplitude and 10 Hz was input into the chamber for 25 min.
- Set (2)–(3) as a cycle, and repeat this cycle for another 2 times with only increasing the voltage gradually to 0.5 and 0.8 Vpp respectively in step (3).
- After finishing the cycles, the AC filed was turned off and 5 mL control solution was exchanged into the chamber to rinise the excess ions before AC field with increased Vpp was re-input. The final voltage amplitude to form GUVs was around 1.3 Vpp.
- Reduce the frequency to 5 Hz and Vpp to 0.5 for releasing GUVs from ITO surface.

### Characterization

Optical and fluorescence microscopic observations were carried out on a Nikon ECLIPSE Ti-U (Nikon, Japan). The crystal phase was evaluated by an X-ray diffractometer (XRD; D/Max-3c, Maxima; 40 kV, 30 mA). Field-Emission Scanning Electron Microscopy (FE-SEM) images and elements mapping were obtained by Quanta-600 F (FEI, America; 20 kV, 10.1 μA). TEM characterizations were carried out on JEM-2100 (JEOL, Japan; 200 KV, 101.8 μA). FRAP were measured by Olympus Fv-1200 laser confocal microscope, the photobleaching laser wavenumber is 405 nm. The diffusion constant D is calculated by the equation D = *w*^2^/(4*t_1/2_*), where *w* is the radius of the beam, and *t_1/2_* is the half time for the fluorescent intensity recovery.

## Author Contributions

F.T. conducted the measurements. P.Y. and F.T. designed the project. P.Y and F.T. analyzed the data. P.Y. prepared the manuscript with assistance from F.T.

## Supplementary Material

Supplementary Information Supplementary Information
