# Supplementary material for: Ca-Mediated Electroformation of Cell-Sized Lipid Vesicles
Source: Sci Rep. 2015 May 7;5:9839. doi: 10.1038/srep09839 (PMC4423497; doi:10.1038/srep09839)
Supplement: Supplementary Information [file srep09839-s1.pdf]

Supplementary Info-1 for the Article

Ca-Mediated Electroformation of Cell-Sized Lipid Vesicles

Fei Tao, Peng Yang*

Key Laboratory of Applied Surface and Colloid Chemistry, Ministry of Education, School of Chemistry and Chemical Engineering, Shaanxi Normal University, Xi'an 710062, China

* Address correspondence to: yangpeng@snnu.edu.cn

Materials: L-α-phosphatidylcholine (egg-PC) was obtained from Sigma-Aldrich (America). Egg-PC was dissolved in chloroform (from Aladdin, purity ≥ 99.9%) to form 2 mg/ml lipid solution. Calcium chloride ($CaCl_2$) was obtained from Tianjin Zhi Yuan Reagent Co., Ltd (China). Sodium carbonate (Na_2CO_3) was obtained from Tianjin Chemical Reagent Co., Ltd (China). Ammonium bicarbonate (NH_4HCO_3) was obtained from Beijing Chemical Reagent Co., Ltd (China). Both sucrose and glucose were obtained from Tianjin Tian Li Chemical Reagent Co., Ltd (China). Magnesium chloride ($MgCl_2$), sodium chloride (NaCl) and Potassium (KCl) chloride were obtained from Kemral Reagent Co., Ltd (China). All chemical reagents were analytical grade. Glass slides coated with indium tin oxide (ITO, sheet resistance from 15 to 27 Ω) were purchased from Shenzhen NanBo Display Technology Co., Ltd (China). For observation of the vesicles with fluorescence microscopy, the following dyes were used: 1,1'-dioctadecyl-3,3,3',3'-tetramethylindocarbocyanine perchlorate (Dil) (excitation at 551 nm and emission at 569 nm) or 3,3'-dioctadecyloxacarbocyanine perchlorate (DiO) (excitation at 440 nm and emission at 450 nm) from Beyotime was added to the lipid solution at concentration 0.1 mol%. All water used in the experiments was processed by Milli-Q system (Milli-Q Advantage A10).

The Hydrophilic Modification on ITO Surface. The detailed description for this method could be referred elsewhere.[1] A thin layer of an aqueous ammonium persulfate solution (30 wt %) was sandwiched between two films (BOPP as the top film, while ITO-coated polyester as the bottom film) and exposed to UV irradiation (high-pressure mercury lamp, 1000 W; UV intensity, 8000 um/cm^2; irradiation time, 120 s). After the irradiation, the films were separated and washed sequentially by copious water, and dried at ambient condition.

Electroformation of GUVs in the Presence of Mg^{2+}, Na^+ and K^+. The main experimental steps were the same as those adopted for the Ca^{2+} adsorption layer. The only variation from the experiments for Mg^{2+}, Na^+, K^+ was the final voltage in the electroformation, which was 0.8 Vpp for Mg^{2+}, 1.8 Vpp for Na^+ and 0.5 Vpp for K^+ respectively.

The Removal of the $CaCO_3$ Mineral Layer from GUVs. The GUVs with $CaCO_3$ mineral layer attached was firstly grown in the electroformation chamber. For a complete removal of the mineral layer around GUVs, a 50 mM sucrose solution (5 ml) at pH 2 (adjusted by 0.1 M HCl) was exchanged into the chamber at 6 rmp by a syringe pump for two times. After that, a low frequency (5 Hz) AC field with 0.5 Vpp was input to the chamber for 25 min to release GUVs from ITO surface.

Scheme S1. The experimental outline to prepare monodispersed GUVs by the calcium adsorption layer models. (1) Pre-step: a swollen multilayer lipid film with phosphate headgroups exposing outwards was formed on ITO surface by appling the AC field at 0.1 Vpp for 25 min. (2) Cycle steps: the uniform calcium adsorption layer was built on the lipid film prepared by the step (1). (3) Vpp-increased steps: the Vpp was increased to a suitable value for forming monodispersed GUVs.

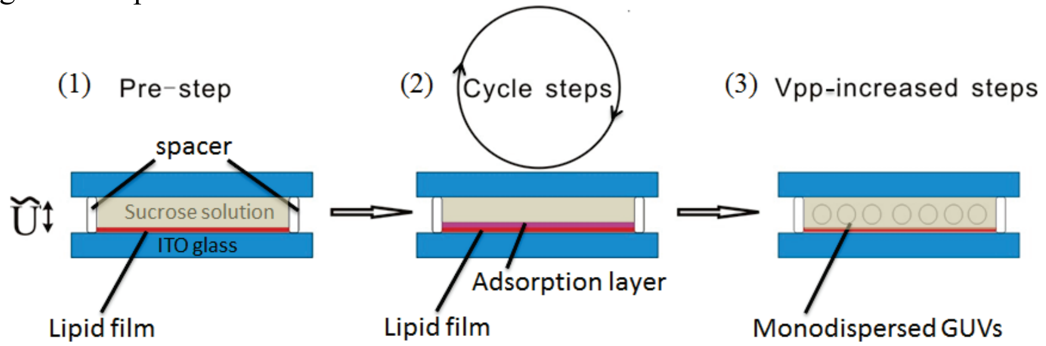

Scheme S2. Schematic representation of the experimental cycles for the electroformation of GUVs mediated by the model of ions adsorption layers. A. Keeping AC field off and exchanging a $CaCl_2$ solution into the chamber by a programmable syringe pump; B. exchanging a control solution into the chamber by a programmable syringe pump; C. a sine AC field was applied between the two ITO electrodes with an increased amplitude. Step A, B and C were repeated as a cycle and three cycles were carried out one by one at 0.3, 0.5 and 0.8 Vpp sequentially.

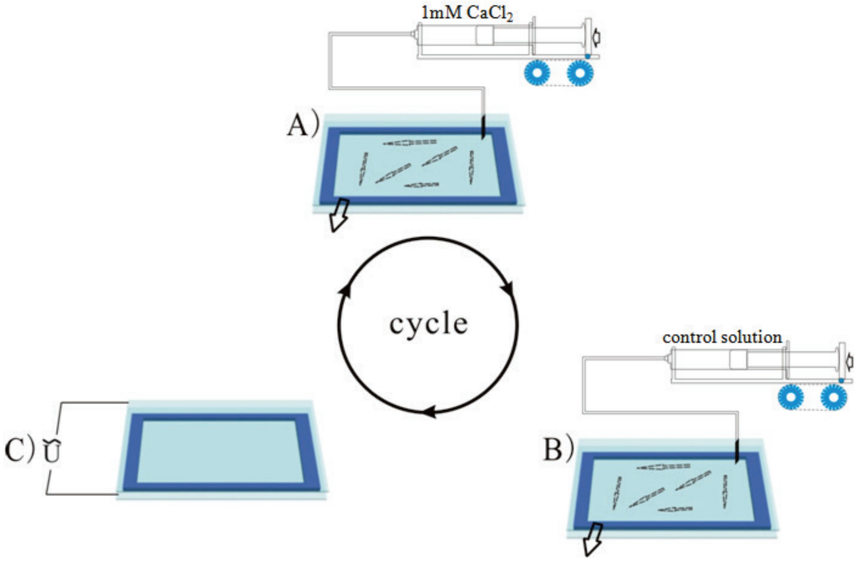

Scheme S3. Schematic representation of the experimental cycles to fabricate the $CaCO_3$ array on the Ca^{2+}-impregnanted lipd film surface. A). Keeping AC field off and exchanging a $CaCl_2$ solution into the chamber by a programmable syringe pump; B). exchanging a Na_2CO_3 solution into the chamber by a programmable syringe pump; C). a sine AC field was applied between the two ITO electrode surfaces with an increased amplitude; D). turning off AC field and exchanging a control solution into chamber to rinse the excess ions. Step A)-D) were repeated as a cycle and three cycles were carried out one by one at 0.3, 0.5 and 0.8 Vpp sequentially.

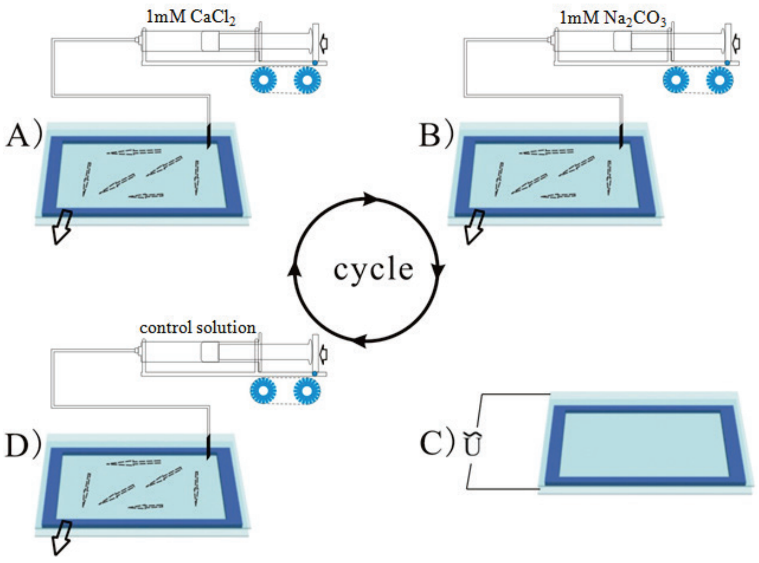

Scheme S4. Schematic representation of the experimental cycles for the electroformation of GUVs mediated by the model of minerals adsorption layer. A). Befor assembling the chamber, a $Ca(OH)_2$ sol was spin-coated twice on the lipid film coated onto the ITO glass; B). a sine AC field was applied between the two ITO electrode surfaces; C). turning off the AC field and exchanging a NH_4HCO_3 solution into the chamber by a programmable syringe pump. Step B and C were repeated as a cycle and four cycles were carried out one by one at 0.1, 0.3, 0.5 and 0.8 Vpp sequentially.

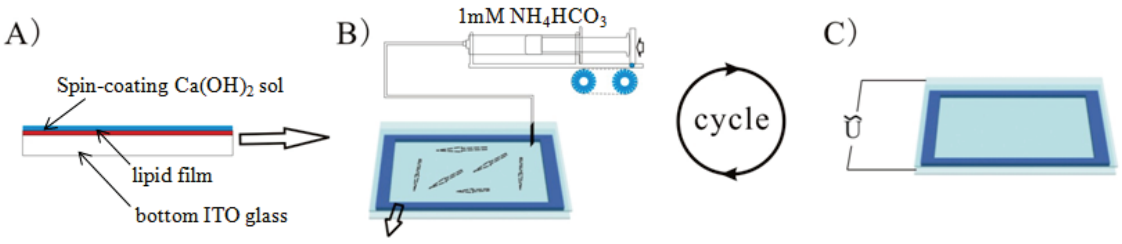

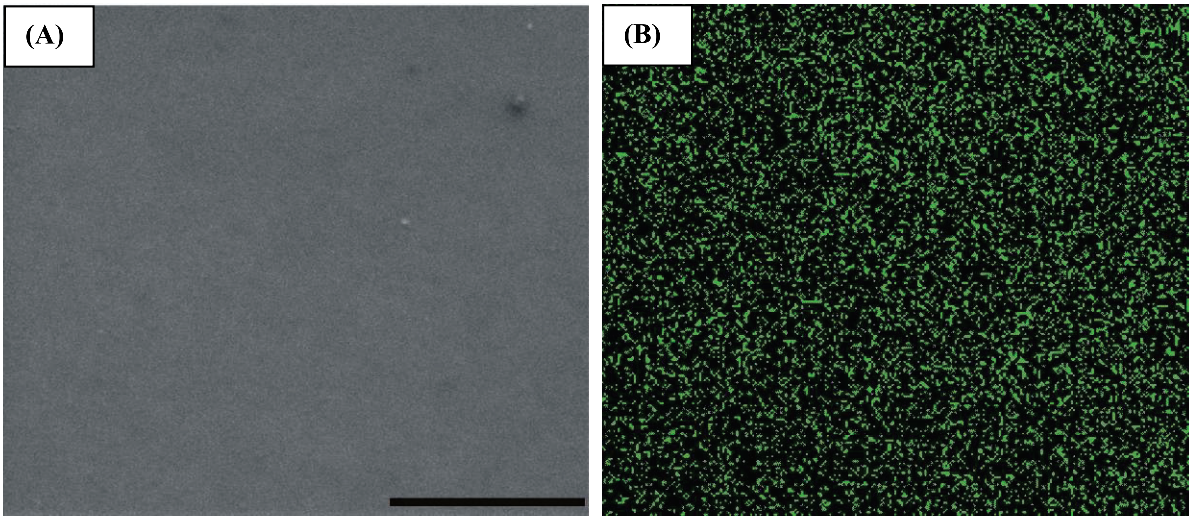

Figure S1. The SEM image for the lipid film (A) and EDS mapping of Ca (B) on the lipid film-coated ITO electrode surface after exchanging the 1 mM $CaCl_2$ solution and the control solution for the construction of Ca^{2+} adsorption layer. The Ca element mapping showed a large amount of Ca^{2+} retained on the lipid film after the control solution flushing the chamber, which implied the Ca^{2+} adsorption layer existed robustly on the film surface. Scale bar =200 μm.

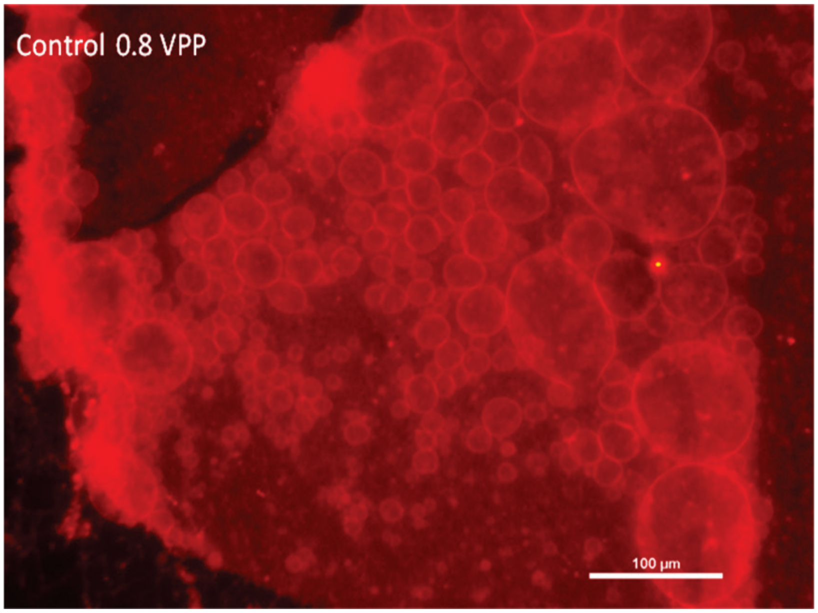

Figure S2. The fluorescent image for the conventional electroformation of GUVs at 0.8 Vpp amplitude without any confinements applied. The size statistics of this GUVs population was shown in Figure 2 of the main text.

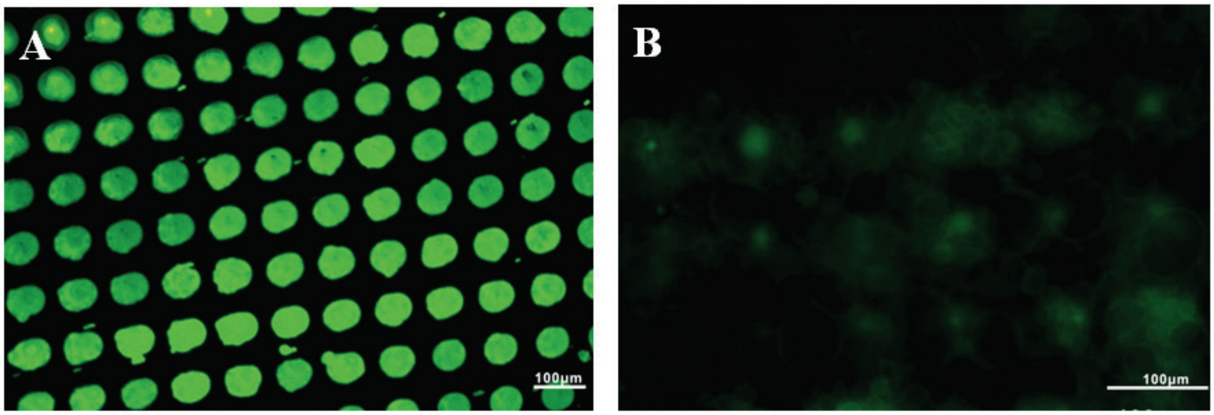

Figure S3. The fluorescent images for GUVs prepared by the combination of electroformation with microcontact printing: A) a micropatterning of the lipid film doped with DiO fluorescein onto ITO surface by microcontact printing; B) the resultant GUVs by the electroformation in the chamber assembled from the ITO shown in (A). The growing solution was 50 mM sucrose solution, and the GUVs were seen to sprout from the pattern after applying AC field for 1 hr.

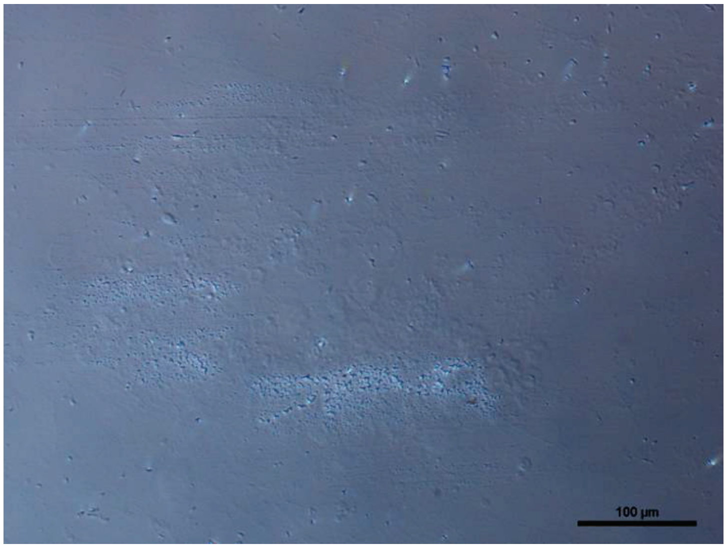

Figure S4. The DIC image on the Ca^{2+}-impregnated lipid film after exchanging the Na_2CO_3 and control solution into the chamber. In this case, instead of a gradual increase on the voltage power via multiple cycles, the amplitude was directly elevated to 0.8 Vpp without the use of cycles shown in Scheme S3. By this process, there was no $CaCO_3$ array observed.

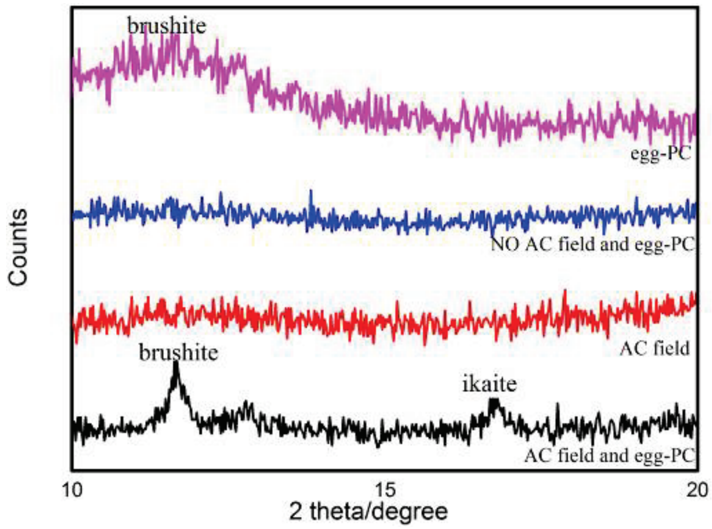

Figure S5. XRD results for the $CaCO_3$ fabrication on the lipid film-coated ITO surfaces after contacting the surfaces with the Ca^{2+} solution at different conditions: purple curve represented the deposition of Ca^{2+} on the lipid film-coated ITO surface without AC field applied; bule curve represented the deposition of Ca^{2+} directly on bare ITO surface without the lipid film coated and AC field applied; red curve represented the deposition of Ca^{2+} directly on bare ITO surface without the lipid film coated but with AC field applied during the deposition; black curve represented that the the deposition of Ca^{2+} on the lipid film-coated ITO surface with AC field applied during the deposition. In four curves, only black curve presented the clear peaks assigned to brushite and ikaite, indicating the valitidy of our hypothesis that the formation of calcite mineral was mainly controlled by the lipid film and AC field.

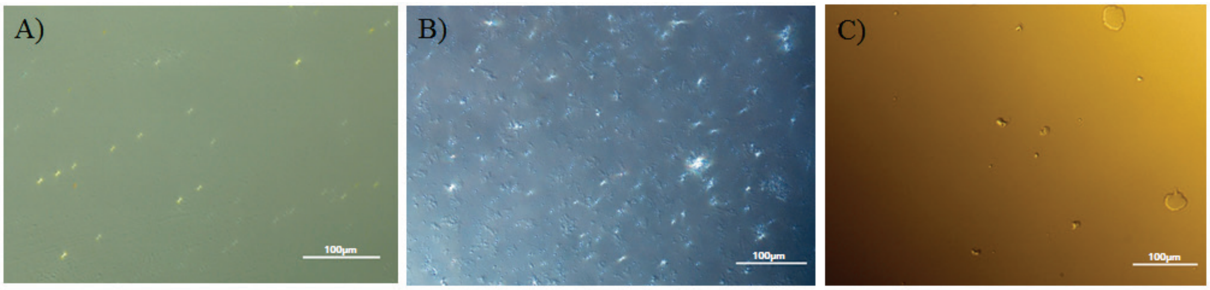

Figure S6. The DIC images for the control samples obtained by the processes to transform Ca^{2+} to $CaCO_3$ under different conditions. (A) the use of bare ITO surface with AC field applied; (B) the use of the lipid film-coated ITO surface without AC field applied; (C) the use of bare ITO surface without AC field applied. Only image (A) showed some ordered crystal array, and in contrast, the image (B) showed randomly distributed precipitations and the image (C) did not present obvious formation of crystalline particles. This comparison clearly indicated that the formation of the ordered calcite mineral as mentioned in the main text was largely driven by the AC field-mediated Ca^{2+} binding with lipid headgroups and subsequent transformation to calcite minerals.

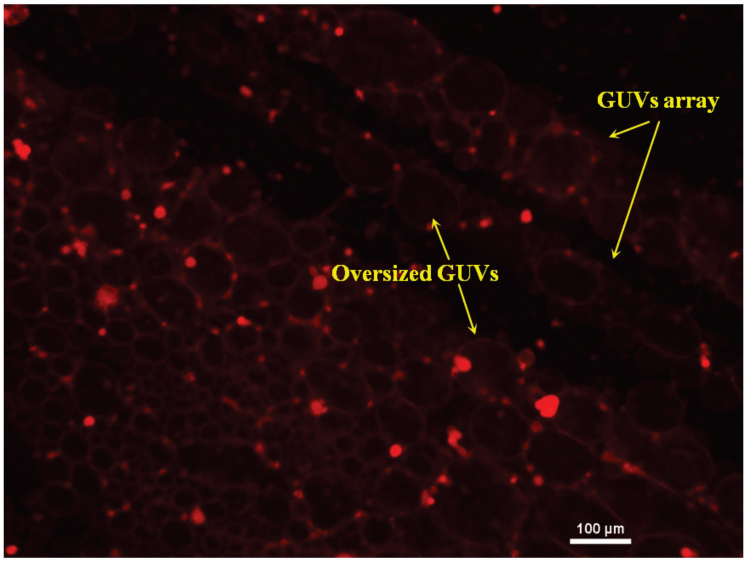

Figure S7. The fluorescent image for GUVs formation during the gradual transformation of Ca^{2+} ions to $CaCO_3$ minerals. The restriction role on the growth of GUVs under AC field was getting worse with the transformation proceeding, because as observed in this figure, besides the formation of GUVs array (as indicated by the arrow at upper right), some over-sized (>100 μm) GUVs were also emerged (as indicated by the arrow at lower left).

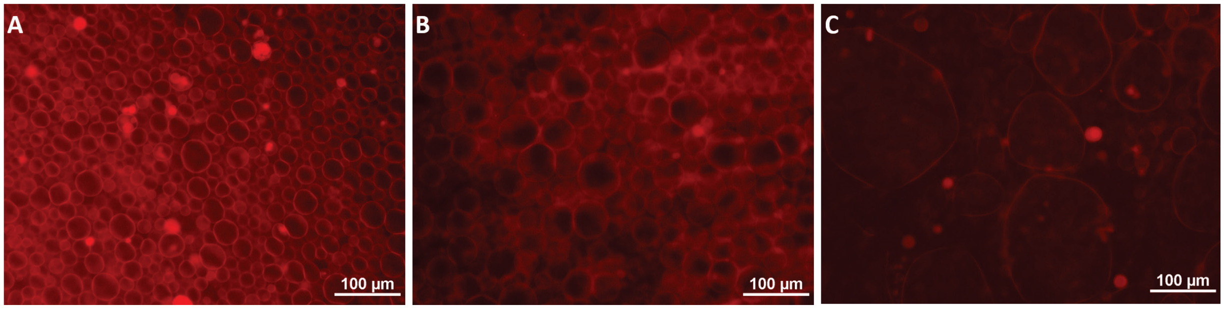

Figure S8. The fluorescent images of GUVs formed in the presence of Mg^{2+} (A), Na^{+} (B), and K^{+} (C) respectively.

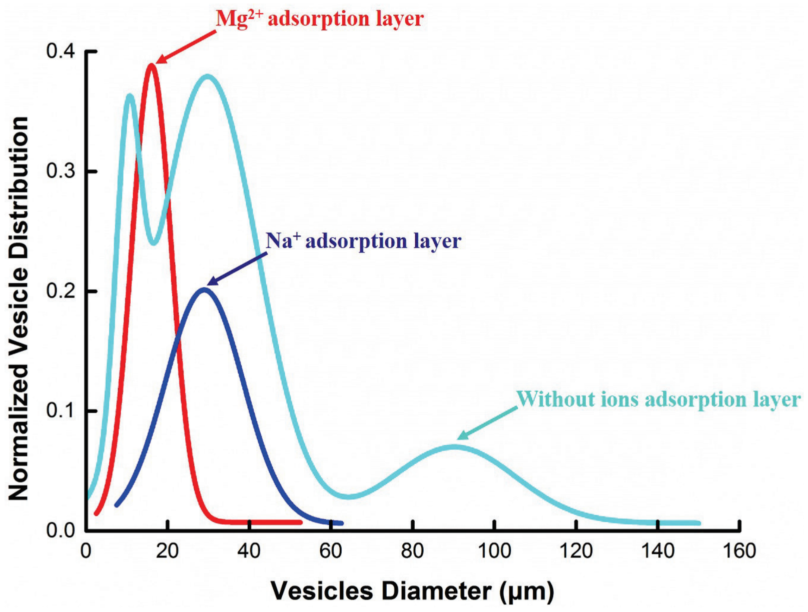

Figure S9. The size distribution of GUVs mediated by Mg^{2+} and Na^{+} adsorption layers respectively. For clarity, the distribution curve obtained by the conventional electroformation without any ions adsorption layer was also included as represented by the blue line.

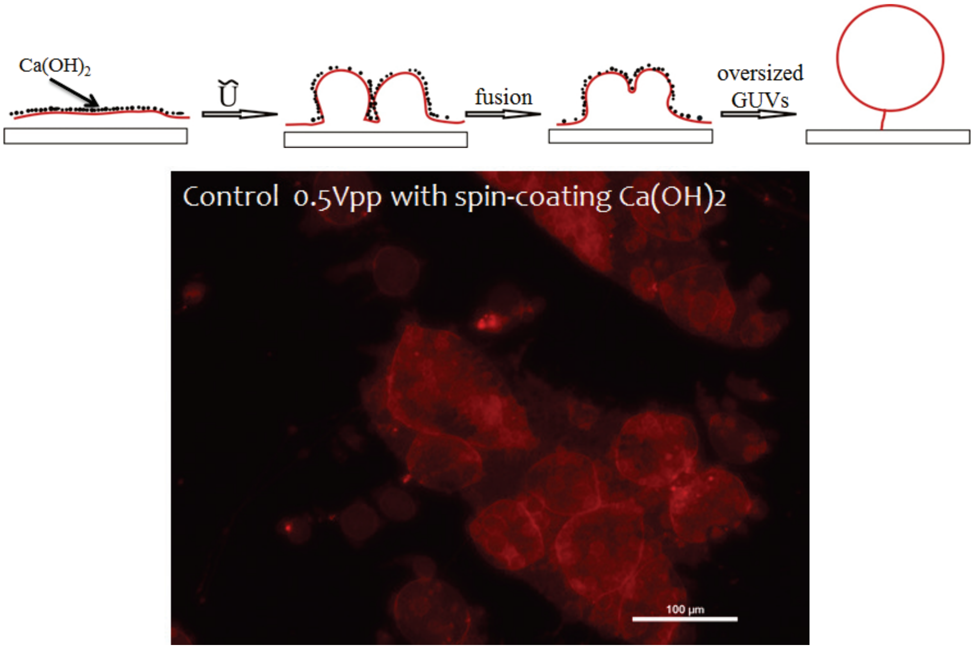

Figure S10. The schematic process (top) and corresponding fluorescent image (bottom) for the $Ca(OH)_2$ sol-induced formation of oversized vesicles and aggregated vesicles. In this case, the step of exchanging the NH_4HCO_3 solution into the chamber for the mineralization of $Ca(OH)_2$ was excluded, and thereby, the restriction function from the calcite mineral was lost in the system, resulting in the oversized and aggregated GUVs.

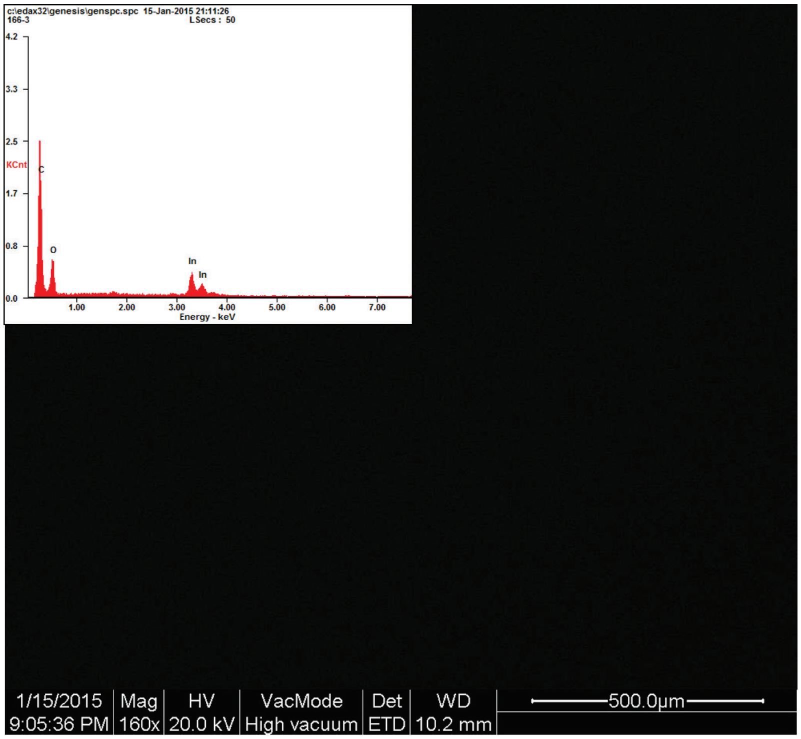

Figure S11. The SEM and Energy Dispersive Spectrometer (EDS) analysis (inset) of the lipid membrane after the complete dissolution of the attached $CaCO_3$ mineral layer. From the EDS, there was no signal assigned to calcium element found, indicating the effective removal of the attached $CaCO_3$ mineral layer.

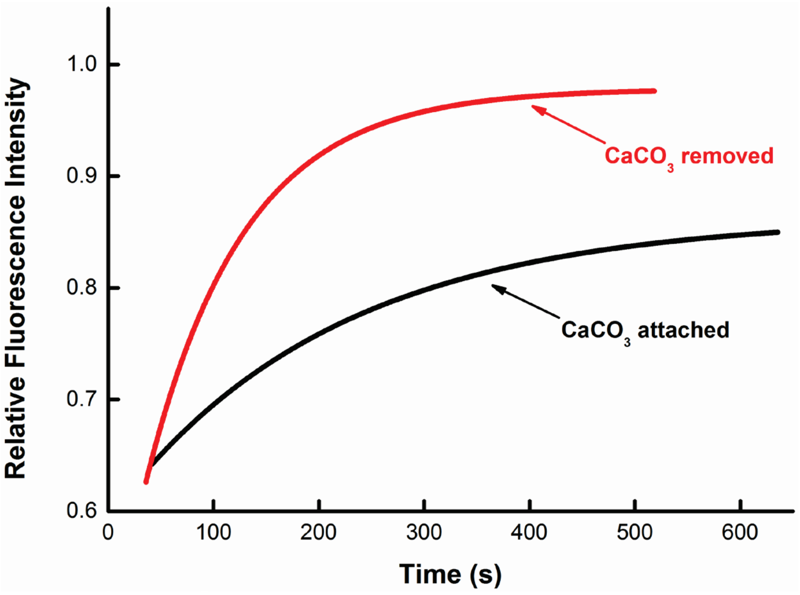

Figure S12. The FRAP (Fluorescence Recovery After Photobleaching) curves of the lipid membrane with (black curve) or without (red curve) the $CaCO_3$ mineral layer attached.

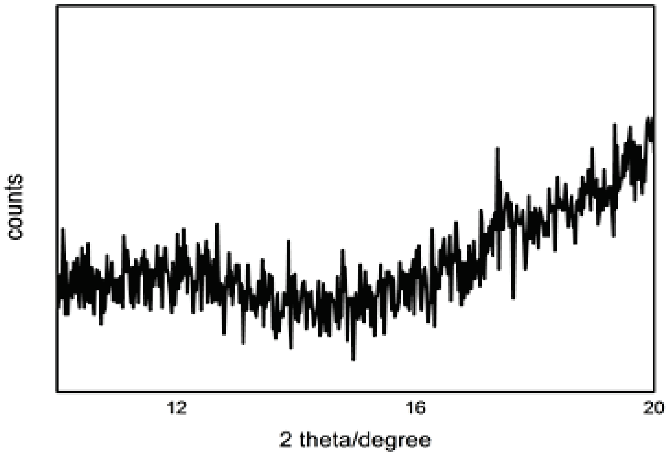

Figure S13. The XRD result on the lipid film surface spin-coated by $Ca(OH)_2$ sol. No peaks assigned to brushite were observed.

Table S1. The solubility coefficients of calcium phosphate, magnesium phosphate, sodium phosphate and potassium phosphate at 20℃.

	$Mg_3(PO_4)_2$	$Ca_3(PO_4)_2$	Na_3PO_4	K_3PO_4
Solubility coefficient	$2.58*10^{-4}$	$2*10^{-3}$	12.1	92.3

(This data from Wikipedia)

Reference

[1] Mu X. Y, Guo S. L. and Yang P. Modification of indium tin oxide with persulfate-based photochemistry toward facile, rapid, and low-temperature interface-mediated multicomponent assembling. *Langmuir* **30,** 4945-4951(2014).

Supplementary Info-2 for the Article

Ca-Mediated Electroformation of Cell-Sized Lipid Vesicles

Fei Tao, Peng Yang*

Key Laboratory of Applied Surface and Colloid Chemistry, Ministry of Education, School of Chemistry and Chemical Engineering, Shaanxi Normal University, Xi'an 710062, China

* Address correspondence to: yangpeng@snnu.edu.cn

To describe the whole process which contains the stable Ca^{2+} Stern layer formed on the lipid film and non-uniform concentration polarization of Ca^{2+} adsorption layer under AC electric field, the Electric Double Layer (Part Ⅰ) and Electrokinetic theory (Part II) are applied:

Part I. Electric Double Layer [1]

Without AC electric field applied, Ca^{2+} on the lipid film surface forms a Stern plane. By the use of Electric Double Layer theory, we derive that in such Stern plane, Ca^{2+} follows the Boltzmann distribution as eq. (1).

$$n_i = n_i^r \exp\left(-z_i e\varphi / k_b T\right) \quad (1)$$

Where $z_i e$ is the charge of the ith ionic species, φ is the electric potential, k_b is the Boltzmann's constant, T is the temperature, n_i^r is the Ca ion number density at the point *r* on the lipid film.

Eq. (1) is valid under the electroneutrality condition: $\sigma^0 + \sigma^i + \sigma^d = 0$ (2) where σ^0 is the surface charge, σ^i is the charge of the Stern layer, σ^d is the charge of the diffuse layer.

When some points are far away enough from the surface, the corresponding electric potential φ is unaffected by the presence of charged lipid film surface, *i.e.* $\varphi \rightarrow 0$, and then by eq. (1), the ion number densities $n_i \rightarrow n_r$ (the Ca ion number density at point *r* far away enough from the surface).

Part II. Electrokinetic Theory [2, 3]

Electrokinetic theory is used to derive the distribution of Ca^{2+} and resultant electroosmotic flow across the lipid film surface under AC electric field. Electrokinetic potential (ψ), the ion number densities (n_j), drift velocities (v_j), the fluid velocity (u), and the pressure (p) at every point (r) in the domain are necessary for a description of the lipid film/Ca^{2+} system.

2.1 Poission's equation:

$$\nabla^2 \psi(r,t) = \frac{\rho(r,t)}{\varepsilon} \quad (3)$$

Where $\rho(r,t) = \sum_i Z_j e n_j(r,t)$ (4)

Substitution of eq. (3) into eq. (1) results in the Poisson-Boltzmann equation for electric potential

$$\nabla^2 \varphi(r,t) = -\frac{1}{\varepsilon}\sum_i n_i^r z_i e \exp\left(-z_i e\varphi / k_b T\right) \quad (5)$$

2.2 Navier-Stokes equations:

$$\eta_0 \nabla^2 u - \nabla p = \rho \nabla \psi + \rho_0 \frac{du}{dt} \quad (6)$$

And

$$\nabla \cdot u = 0 \quad (7)$$

Where η_0 and ρ_0 are the viscosity and mass density of the medium.

Under the precondition of conservation of ionic species, eq. (8) is derived from eq. (6):

$$\frac{dn_i}{dt} + \nabla \cdot (n_i v_i) = 0 \quad (8)$$

Under the condition described in eq. (8), eq. (9) is derived by Newton's second law of motion:

$$-\lambda_i (v_i - u) - z_i e \nabla \psi - k_b T \nabla Inn_i = m_i \frac{dv_i}{dt} \quad (9)$$

Where λ_i is the drag coefficient, $z_i e$ is the charge of the ith ionic species, k_b is the Boltzmann's constant, T is the temperature, and m_i is the apparent ionic mass.

2.3 Boundary condition:

Under AC electric field, Electrokinetic equations are not valid for the Stern layer due to the assumption concerning the discreetness of the charge. Therefore, we need the boundary condition at the Slip plane that is a far field type boundary condition as shown in equation:

At $r = a$

$$\psi_0 = \zeta$$
$$u = 0 \quad (10)$$
$$v_j \cdot \hat{n}$$

When applying the boundary condition (10) into eq. (5) and (9), we can derive eq. (11) and (12):

$$\psi \to V_{peak} \sin(\omega t) \cdot \hat{n} \quad (11)$$

and

$$n_j \to n_j^b \text{ as } r \to \infty \quad (12)$$

Where $V_{peak}\sin(\omega t)$ is the applied electric field (sine wave) and n_j^b is the bulk number density of the jth ionic species under AC electric field. Regarding to our case, eq. (11) and (12) indicate that n(Ca^{2+}) as the number density of Ca^{2+} ions and ψ(r, t) as the corresponding electric potential distributed across the lipid film surface are determined by $V_{peak}\sin(\omega t)$, which fits a sine wave distribution (Figure S1). Such nonlinear phenomena are usually classified by origin of the concentration polarization, because Ca ions in Slip plane have non-zero time average velocities in AC electrokincetics at boundary condition.

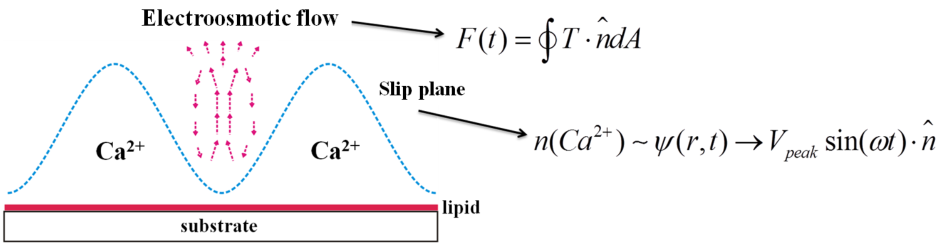

Figure S1. Schematic formation of Ca ions Slip plane under AC electric field because of concentration polarization (a sine wave function) and resultant electroosmotic flow (a circular integral) in areas of C(Ca^{2+})$_{min}$.

2.4 Maxwell stress:

$$F(t) = \oiint T \cdot \hat{n} dA \quad (13)$$

Eq. (13) represents force of electroosmostic flow in areas of C(Ca^{2+})$_{min}$, which is an circular integral (as shown in Figure S1).

T is the second order Maxwell stress tensor

$$T = \varepsilon\left(E \otimes E - \frac{1}{2}E^2 I\right) \quad (14)$$

References

1 Mangelsdorf C. S., White L. R. The dynamic double layer. J. Chem. Soc., Faraday Trans., **94**, 2441-2452 (1988).

2 Mangelsdorf C. S., White L. R. Electrophoretic mobility of a spherical colloidal particle in an oscillating electric field. J. Chem. Soc., Faraday Trans., **88**, 3567-3581 (1992).

3 Verwey E. J. W., Overbeek J. T. G., Nes K. V. Theory of the stability of lyohobic colloids; the interaction of sol particles having an electric double layer (Elsevier Pub. Co. press, 1948).
